# Pyrroloquinoline Quinone (PQQ) as a Mitochondrial Rejuvenation Strategy in Aesthetic Dermatology: Mechanisms, Therapeutic Potential, and Future Clinical Applications

**DOI:** 10.3390/biom16081197

**Published:** 2026-08-17

**Authors:** Kyu-Ho Yi

**Affiliations:** 1 Division in Anatomy & Developmental Biology, Department of Oral Biology, Yonsei University College of Dentistry, 50-1 Yonsei-ro, Seodaemun-gu, Seoul 03722, Republic of Korea; kyuho90@daum.net; 2 You and I Clinic, Seoul 27454, Republic of Korea

**Keywords:** pyrroloquinoline quinone, PQQ, mitochondria, skin aging, photoaging, aesthetic dermatology, oxidative stress, cellular senescence

## Abstract

Background: Mitochondrial dysfunction is increasingly recognized as a central contributor to intrinsic skin aging, photoaging, cellular senescence, impaired extracellular-matrix homeostasis, dysregulated pigmentation, and delayed recovery after energy-based or minimally invasive procedures. Pyrroloquinoline quinone (PQQ) is a redox-active ortho-quinone that has attracted interest because it can participate in repeated redox cycling, protect mitochondrial function, and activate signaling associated with mitochondrial biogenesis. Objective: This narrative review evaluates the mechanistic basis, available dermatologic evidence, translational opportunities, and major uncertainties surrounding PQQ as a mitochondrial rejuvenation strategy in aesthetic dermatology. Methods: PubMed/MEDLINE and Europe PMC were searched from database inception through 10 August 2026 using PQQ-, mitochondrial-, skin-, delivery-, and safety-related terms; reference lists were also screened. Mechanistic, preclinical, skin-focused, human, and regulatory evidence was synthesized narratively. Results: Experimental studies support PQQ-mediated activation of mitochondrial biogenesis pathways and protection against oxidative injury in several cell and animal systems. Skin-specific evidence includes attenuation of oxidative stress, DNA damage, senescence markers, and matrix metalloproteinases in accelerated-aging mouse models; protection of UVA-exposed human dermal fibroblasts; suppression of UVB-induced caspase-1 release in keratinocytes; a small oral dry-skin study; and a multi-ingredient topical study containing an allyl PQQ derivative. These studies do not establish PQQ-specific clinical aesthetic efficacy. Conclusion: PQQ is a biologically plausible mitochondrial-support compound, but it should currently be regarded as an investigational ingredient rather than an established aesthetic treatment. Carefully designed formulation, toxicology, dose-finding, biomarker, and randomized clinical studies are required before claims regarding wrinkle reduction, pigment improvement, enhanced collagen production, or accelerated post-procedure recovery can be justified.

## 1. Introduction

Aesthetic dermatology has traditionally targeted visible endpoints such as wrinkles, laxity, dyspigmentation, erythema, enlarged pores, and uneven texture. Increasingly, these phenotypes are interpreted as downstream manifestations of altered cellular energetics, redox imbalance, chronic low-grade inflammation, senescence, and extracellular-matrix dysregulation. Mitochondria sit at the intersection of these processes. They generate adenosine triphosphate (ATP), coordinate redox signaling, regulate apoptosis and calcium homeostasis, influence innate immunity, and provide metabolites required for biosynthesis and epigenetic regulation. With chronological aging and repeated environmental exposure, mitochondrial DNA accumulates damage, respiratory efficiency declines, reactive oxygen species (ROS) increase, and stress-response pathways become persistently activated. In skin, these changes can reduce keratinocyte renewal, compromise barrier recovery, alter melanocyte behavior, suppress fibroblast matrix production, and increase matrix metalloproteinase (MMP) activity [1,2,3,4,5,6,7,8].

The concept of “mitochondrial rejuvenation” does not imply that a single molecule can reverse biological aging. Rather, it describes interventions intended to improve mitochondrial quantity, quality, stress resistance, or turnover sufficiently to support more youthful cellular function. Current strategies include exercise mimetics, NAD+ precursors, sirtuin-modulating compounds, mitophagy activators, redox-active molecules, and agents that influence the master regulator peroxisome proliferator-activated receptor gamma coactivator 1-alpha (PGC-1α). Within this landscape, pyrroloquinoline quinone (PQQ) is of interest because it combines potent redox activity with signaling effects related to mitochondrial biogenesis [9,10,11,12,13,14,15].

PQQ was first characterized as a bacterial redox cofactor and is present in small amounts in foods and mammalian tissues. It exists in oxidized and reduced states, enabling repeated electron transfer. Although it has sometimes been described as a vitamin-like compound, its status as an essential human vitamin has not been established. The best-supported biological model is that PQQ acts as a bioactive redox modulator and signaling molecule rather than merely as a stoichiometric antioxidant [10,12,15]. Experimental studies demonstrate activation of CREB and PGC-1α, increased expression of nuclear respiratory factors and mitochondrial transcription factor A (TFAM), preservation of mitochondrial membrane potential, and reduced oxidative injury [9,11,13,14,15,16]. These mechanisms are relevant to skin aging, but relevance alone is not proof of clinical efficacy.

This review examines PQQ from an aesthetic-dermatology perspective. It distinguishes established biochemical observations from skin-specific preclinical findings and from proposed future applications. It also addresses formulation, delivery, safety, endpoints, and regulatory considerations that must be resolved before PQQ can be positioned responsibly as a topical cosmeceutical, oral adjunct, intradermal ingredient, or peri-procedural recovery agent.

### Literature Search Strategy

PubMed/MEDLINE and Europe PMC were searched from database inception through 10 August 2026. Search terms combined (“pyrroloquinoline quinone” OR PQQ OR methoxatin) with terms for mitochondria, oxidative stress, skin, dermis, epidermis, fibroblasts, keratinocytes, melanocytes, aging, photoaging, pigmentation, wound healing, aesthetic procedures, pharmacokinetics, bioavailability, safety, and toxicity. Reference lists of eligible papers and recent reviews were screened for additional records.

Peer-reviewed English-language mechanistic studies, original in vitro and animal studies, human trials, and regulatory safety assessments were included when they contained PQQ-specific information relevant to mechanism, cutaneous biology, delivery, pharmacokinetics, or safety. Duplicates, non-peer-reviewed promotional material, studies lacking PQQ-specific data, and papers without a plausible connection to the review scope were excluded. Because the literature is sparse and heterogeneous, the evidence was synthesized as a narrative rather than a systematic review; study design, model, route, dose, and evidentiary limitations were considered when interpreting findings.

## 2. Chemical and Biological Characteristics of PQQ

PQQ is a tricyclic o-quinone with multiple carboxyl groups that confer high polarity and metal-binding capacity. Commercial preparations frequently use the disodium salt to improve handling and water solubility. Its quinone structure allows reversible reduction to pyrroloquinoline quinol, enabling redox cycling. In cell-free systems, this cycling can support catalytic antioxidant behavior, but the biological outcome depends on concentration, local reducing equivalents, metal ions, oxygen tension, and cellular compartment. As with other quinones, a compound that is protective at one concentration can theoretically become pro-oxidant under another set of conditions. This concentration dependence is a central translational issue for skin formulations and injectable concepts [10,12,16,17].

Chemically, free PQQ is 4,5-dioxo-4,5-dihydro-1H-pyrrolo [2,3-f]quinoline-2,7,9-tricarboxylic acid (C14H6N2O8). Its two-electron redox pair can be represented as PQQox + 2e− + 2H+ ⇌ PQQH2. For free PQQ at pH 7.0, a conditional standard potential of −0.175 V versus the saturated calomel electrode was reported; this value is not universal and varies with pH, ionic environment, hydration, metal binding, and protein association [18]. Ascorbate reduces PQQ to PQQH2 in pH 7.4 buffer, after which PQQH2 can be reoxidized by oxygen [19]. These observations identify ascorbate as a demonstrated reductant in a defined biochemical system. The relative contributions of NAD(P)H- and glutathione-dependent systems in cutaneous cells remain uncertain, and unrestricted intracellular redox cycling should not be assumed.

PQQ is not simply a direct ROS scavenger. Its more durable effects appear to arise from modulation of gene expression and mitochondrial signaling. Dietary deficiency experiments in rodents have been associated with reduced mitochondrial content, impaired growth, and altered metabolic function, whereas repletion improves mitochondrial indices [20,21,22,23]. In cultured hepatocytes, PQQ increased mitochondrial number and respiratory capacity through CREB phosphorylation and PGC-1α activation. Silencing CREB or PGC-1α attenuated this response, supporting pathway specificity [9]. Other work indicates that PQQ can enhance cellular NAD+ availability and SIRT1 activity, promoting PGC-1α deacetylation and transcriptional function [13].

For dermatologic translation, physicochemical behavior is as important as pathway biology. PQQ is hydrophilic, susceptible to interactions with metal ions, and may be affected by light, pH, oxygen, and excipient composition. A topical product must deliver biologically active PQQ across the stratum corneum while maintaining stability and avoiding irritation. An intradermal formulation would face a much higher safety threshold, requiring pharmaceutical-grade purity, sterility, endotoxin control, compatibility testing, degradation profiling, and local toxicology. Oral supplementation avoids barrier-delivery problems but makes skin exposure indirect and creates uncertainty regarding systemic absorption, metabolism, and the concentration reaching cutaneous tissues.

## 3. Mitochondrial Dysfunction in Cutaneous Aging

Human skin is metabolically heterogeneous. Basal keratinocytes proliferate and differentiate to maintain the epidermis; fibroblasts synthesize and remodel matrix; melanocytes produce pigment; endothelial cells regulate perfusion; immune cells survey barrier integrity; and adipocytes contribute endocrine and structural functions. Each cell population has a distinct mitochondrial demand. Aesthetic aging arises not from one mitochondrial defect but from interacting changes in respiratory capacity, redox signaling, mitochondrial dynamics, mitophagy, and mitochondrial-to-nuclear communication [1,2,3,4,5,6,7,8,24,25,26,27,28].

Ultraviolet radiation is a major accelerator of mitochondrial dysfunction. UVA penetrates deeply and generates oxidative stress in epidermal and dermal cells. Mitochondrial ROS activate AP-1 and NF-κB pathways, inducing MMPs and inflammatory mediators while suppressing procollagen synthesis. Repeated exposure can create clonally expanded mitochondrial DNA deletions in photoexposed skin. These changes are not merely biomarkers; they contribute to altered matrix turnover and persistent stress signaling [2,5,6,29,30,31,32,33]. Pollution, tobacco smoke, visible light, infrared radiation, and heat may add to this burden through oxidative and inflammatory mechanisms [34,35,36,37,38].

Chronological aging is associated with reduced respiratory-chain efficiency, altered mitochondrial morphology, impaired mitophagy, and accumulation of senescent cells. Senescent fibroblasts remain metabolically active but secrete inflammatory cytokines, chemokines, growth factors, and proteases collectively known as the senescence-associated secretory phenotype (SASP). The SASP can spread dysfunction to neighboring cells and promote matrix degradation. Mitochondria are important regulators of senescence, and mitochondrial dysfunction-associated senescence can differ from classic DNA-damage-induced senescence in its secretory profile [25,26,27,28,39,40,41,42].

The epidermal barrier also depends on adequate energy and redox control. Keratinocyte differentiation, lipid processing, tight-junction function, and repair after injury require coordinated metabolism. Mitochondrial ROS can be physiological signals, but excessive or prolonged ROS may activate inflammasomes, impair differentiation, and prolong inflammation. In melanocytes, mitochondria influence oxidative stress responses and may modify melanogenesis indirectly through cellular redox state, calcium signaling, and interactions with keratinocytes. In endothelial cells, mitochondrial dysfunction contributes to impaired nitric oxide signaling and inflammatory activation. These connections make mitochondria a plausible common target for skin quality, although each phenotype requires direct evidence rather than extrapolation.

## 4. Mechanisms Relevant to PQQ-Mediated Mitochondrial Rejuvenation

### 4.1. CREB-PGC-1α Signaling and Mitochondrial Biogenesis

The most cited mechanistic evidence for PQQ is its ability to stimulate mitochondrial biogenesis. Chowanadisai and colleagues demonstrated that PQQ induced phosphorylation of cAMP response element-binding protein (CREB) at Ser133, increased PGC-1α promoter activity, and raised PGC-1α expression. Downstream changes included activation of nuclear respiratory factors and increased TFAM and mitochondrial transcription-related genes [9]. PGC-1α coordinates nuclear programs involved in oxidative metabolism, antioxidant defense, fatty-acid oxidation, and mitochondrial replication. In skin cells exposed to chronic stress, restoration of this program could theoretically improve ATP generation and resilience. However, the magnitude of PGC-1α activation required for benefit, and whether prolonged activation could have undesirable effects, remain uncertain.

### 4.2. NAD+–SIRT1–PGC-1α Coupling

PQQ has also been reported to increase cellular NAD+ and activate SIRT1-dependent deacetylation of PGC-1α [13]. This is conceptually relevant because NAD+ declines with aging and stress, and SIRT1 regulates mitochondrial adaptation, DNA repair, inflammation, and senescence. PQQ therefore may overlap mechanistically with nicotinamide riboside, nicotinamide mononucleotide, caloric-restriction mimetics, and exercise. Combination strategies should not be assumed to be additive. Excessive pathway stacking may produce no benefit, alter redox balance, or increase adverse reactions. Comparative and factorial experiments would be required.

### 4.3. Redox Buffering and Mitochondrial Membrane Preservation

Biochemical and cellular studies indicate that PQQ can preserve mitochondrial membrane potential, limit lipid peroxidation, and attenuate oxidative injury [14,15,16]. In a skin-relevant model, PQQ reduced UVA-associated senescence and apoptosis-related changes in human dermal fibroblasts [43]. Its redox cycling may allow repeated antioxidant action, but cellular protection probably reflects both direct chemistry and indirect induction of endogenous defense systems. For aesthetic dermatology, the key question is whether these effects occur at concentrations achievable in viable epidermis or dermis without cytotoxicity.

### 4.4. Mitochondrial Integrity and Apoptosis

Loss of mitochondrial membrane potential and activation of mitochondria-dependent apoptosis are central consequences of cutaneous oxidative stress. In UVA-exposed human dermal fibroblasts, PQQ attenuated senescence- and apoptosis-related changes through a SIRT1/NRF2/HO-1-associated pathway [43]. This skin-cell evidence is more directly relevant to aesthetic dermatology than organ-specific extrapolation, but it remains an in vitro observation; effective cutaneous exposure, dose-response relationships, and clinical benefit have not yet been established.

### 4.5. Inflammatory Signaling and the Inflammasome

Mitochondrial ROS and mitochondrial damage-associated signals can activate the NLRP3 inflammasome, leading to caspase-1 activation and maturation of IL-1β and IL-18. A skin-focused study of PQQ disodium reported suppression of UVB-induced active caspase-1 release in normal human epidermal keratinocytes, although mitochondrial biogenesis was not increased in the tested skin-cell system [44]. This negative biogenesis result is important: PQQ effects are cell-type, dose, and context dependent. It suggests that anti-inflammatory activity may occur independently of measurable mitochondrial proliferation.

### 4.6. Cellular Senescence and DNA Damage

**Figure 1:**
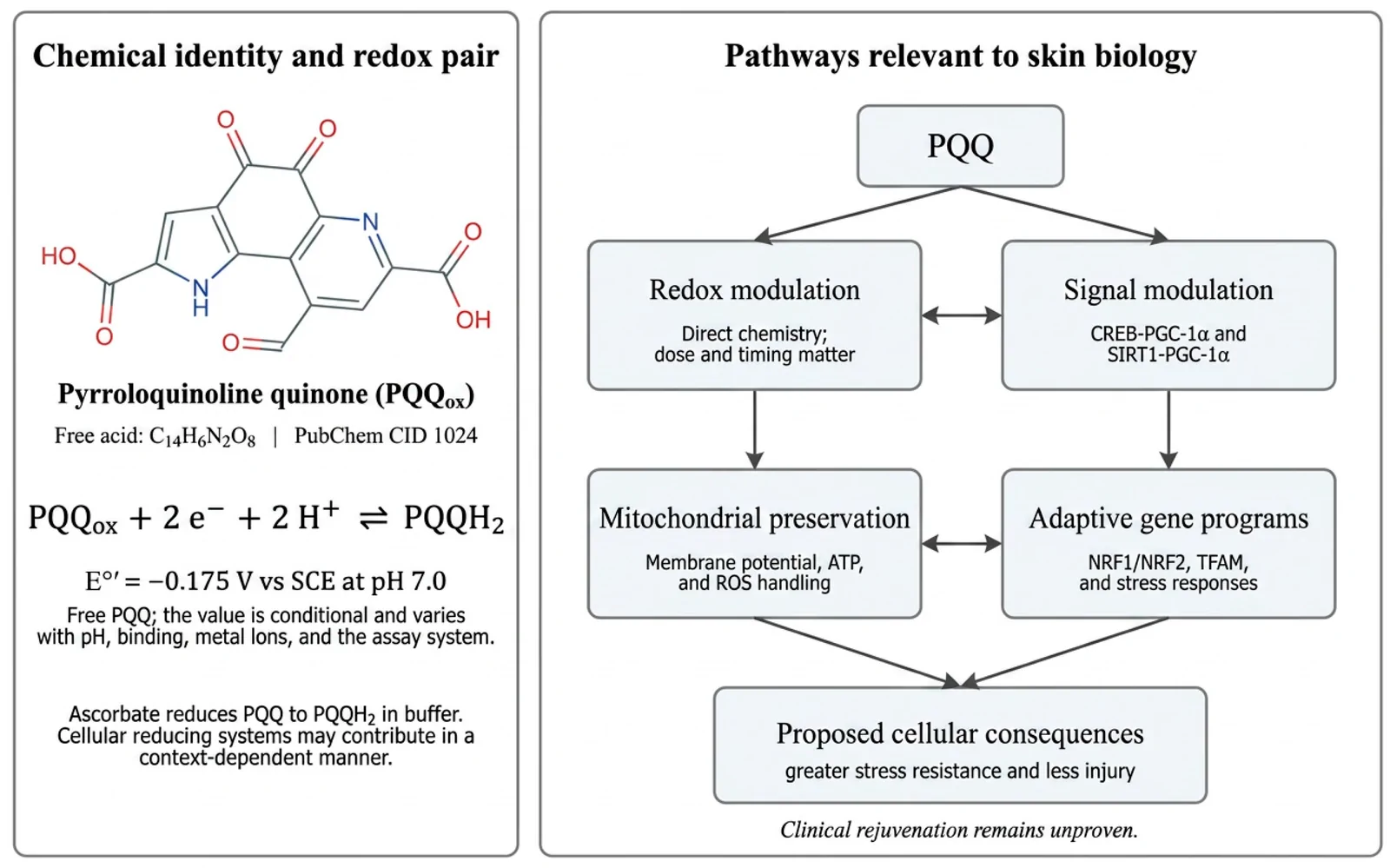
Redox chemistry and proposed mitochondrial-signaling framework for PQQ. The corrected free-acid structure of PQQ (C14H6N2O8) and the PQQox/PQQH2 couple are shown together with the conditional E°′ reported for free PQQ at pH 7.0 [18]. Ascorbate-mediated reduction in buffer has been demonstrated [19]. In the downstream scheme, PQQ is shown only as an abbreviation to avoid implying a specific respiratory-chain interaction. The previous respiratory-complex labels and proton-flow arrows were removed because they were not required to support the proposed pathways. This is an original figure created by the author for this review using the verified PubChem PQQ structure (CID 1024); it is not reproduced or adapted from another publication. The pathways remain mechanistic hypotheses and do not demonstrate clinical skin rejuvenation.

In Bmi-1-deficient mice, a model characterized by oxidative stress and premature aging, oral PQQ reduced cutaneous oxidative stress, DNA damage, senescence, and MMP expression [45]. In an oxidative stress-induced premature senescence model of HEI-OC1 auditory cells, PQQ preserved mitochondrial respiratory function and was associated with SIRT1-PGC-1α signaling [46]. These findings support investigation in photoaged skin and after controlled procedural injury. They do not establish that PQQ is senolytic; rather, it may reduce senescence induction or improve stress tolerance. Distinguishing senomorphic, cytoprotective, and true senolytic effects will be essential (Figure 1).

## 5. Skin-Specific Evidence

### 5.1. In Vitro Studies in Skin-Relevant Cell Models

In vitro studies provide the most direct skin-cell evidence. In UVA-exposed human dermal fibroblasts, PQQ attenuated senescence- and apoptosis-related changes through a SIRT1/NRF2/HO-1-associated pathway [43]. In normal human epidermal keratinocytes, PQQ disodium suppressed UVB-induced active caspase-1 release, but did not increase mitochondrial biogenesis in the tested keratinocytes or fibroblasts [44]. This negative result is important because it suggests that anti-inflammatory or mitochondria-preserving activity may occur without detectable mitochondrial proliferation. A murine B16 melanoma-cell study reported reduced melanogenesis and tyrosinase expression [47], but this model cannot establish efficacy or safety for human pigment disorders. No PQQ-specific three-dimensional human skin-equivalent or ex vivo facial-skin study was identified.

### 5.2. Animal Models Relevant to Skin Aging and Repair

Animal evidence remains limited but is no longer confined to a single accelerated-senescence model. In Bmi-1-deficient mice, oral PQQ reduced cutaneous oxidative stress, DNA damage, senescence, and matrix metalloproteinase expression [45]. A 2025 Bmi-1 knockout study reported improvements in skin morphology, collagen-related measures, proliferation, cell-cycle markers, and autophagy-associated findings [48]. In a compromised-barrier dry-skin mouse model, dietary PQQ for 6 weeks reduced dermal mast cells and epidermal CD3+ T cells [49]. In naturally aged C57BL/6J mice, dietary PQQ improved integument and coat condition, although skin was not the primary mechanistic endpoint [50]. A 2025 excisional-wound study reported accelerated closure and favorable oxidative-stress and histologic findings in mice [51]. The wound model is relevant to repair biology but is not equivalent to facial photoaging or recovery after an aesthetic procedure. The targeted search did not identify a PQQ-specific rat photoaging model or a large-animal model of facial skin aging; this absence is a research gap and should not be filled by extrapolation from non-cutaneous organs.

### 5.3. Existing Human Clinical Evidence

Human evidence is small and heterogeneous. In a small study of healthy women with subjective dry skin, oral PQQ at 20 mg/day for 8 weeks limited the increase in forearm transepidermal water loss and produced favorable questionnaire responses; it did not establish effects on wrinkles, elasticity, pigmentation, or dermal mitochondrial function [49]. In an uncontrolled 12-week study of 40 subjects with mild-to-moderate photodamage, a multi-ingredient topical product containing an allyl PQQ derivative was associated with improvements from baseline and favorable histologic changes [52]. Because the formulation contained multiple active ingredients and lacked a PQQ-only or vehicle-controlled arm, the specific contribution of PQQ cannot be determined.

**Figure 2:**
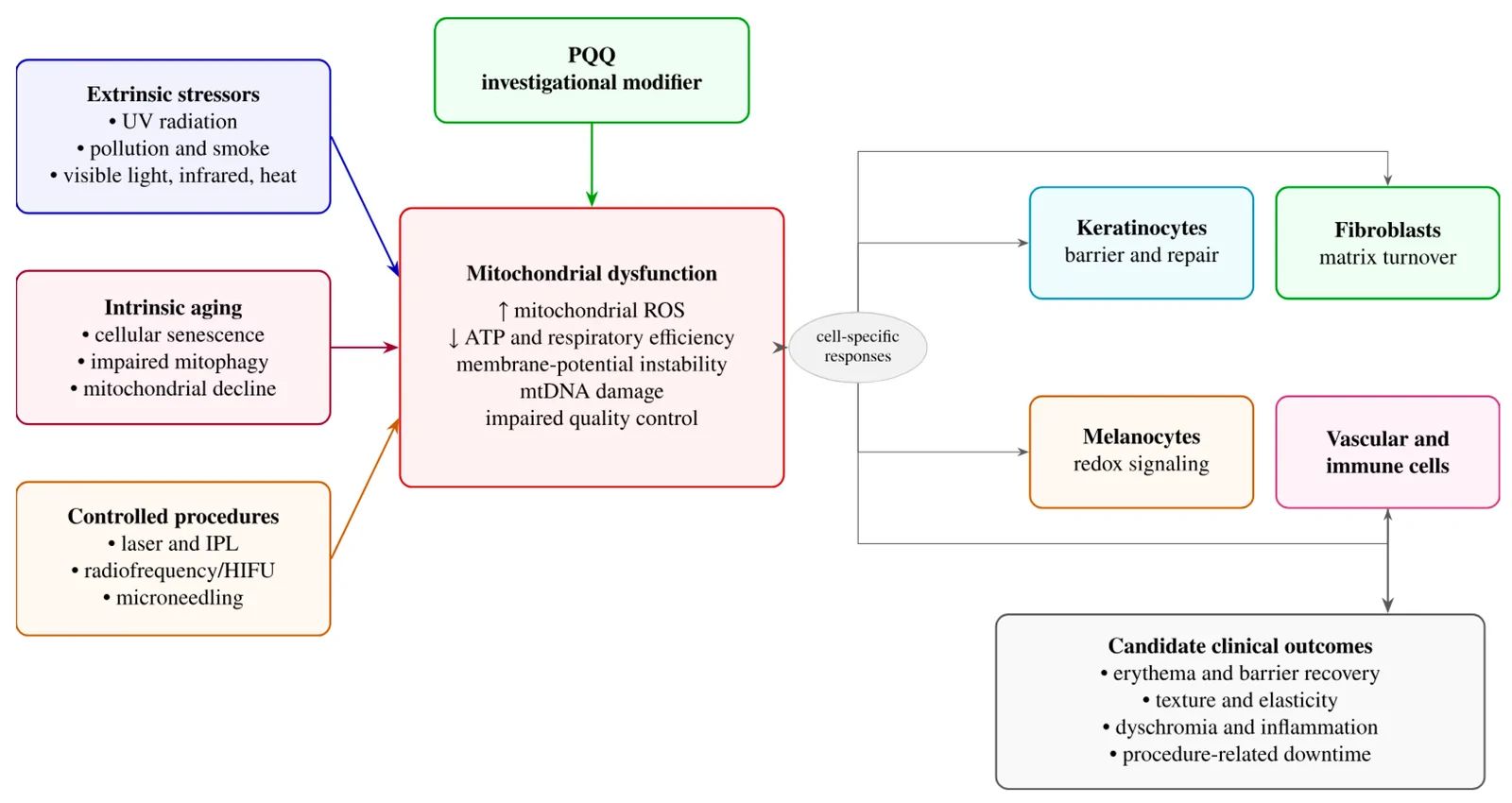
Mitochondrial dysfunction as a convergent target in aesthetic dermatology. Extrinsic stressors, intrinsic aging, and controlled procedural injury can converge on mitochondrial dysfunction. Consequences in keratinocytes, fibroblasts, melanocytes, endothelial cells, and immune cells may contribute to barrier impairment, matrix degradation, dyschromia, inflammation, and delayed recovery. PQQ is proposed as a modifier of this central stress node, but clinical validation is required. This is an original figure created by the author for this review; it is not reproduced or adapted from another publication. ↑ is “increased”; ↓ is “decreased”.

Human supplementation studies outside dermatology and regulatory assessments provide short-term exposure and safety information, commonly around 20 mg/day, but do not validate a skin-rejuvenation dose [53,54,55,56,57,58]. Accordingly, the evidence hierarchy is robust biochemical plausibility; moderate experimental evidence for mitochondrial protection in diverse models; limited skin-specific cell and mouse evidence; small, potentially confounded human skin studies; and no high-quality PQQ-specific randomized trial for aesthetic endpoints or post-procedure recovery. This hierarchy should govern scientific claims and study design (Figure 2).

## 6. Potential Applications in Aesthetic Dermatology

### 6.1. Topical Anti-Aging Formulations

A topical PQQ product could be positioned to support mitochondrial resilience against environmental stress rather than to promise direct rejuvenation. Candidate endpoints include reduction in oxidative biomarkers, improved barrier recovery, decreased erythema after standardized UV or irritant challenge, and long-term changes in fine lines or texture. Formulation obstacles include hydrophilicity, stratum-corneum penetration, photostability, metal interactions, and concentration-dependent irritation. Encapsulation in liposomes, polymeric nanoparticles, hydrogels, or dissolving microneedles may improve delivery, but each platform changes regulatory and safety requirements. A published study of a multi-ingredient topical antioxidant containing an allyl PQQ derivative reported improvements in photodamaged skin, but the combination formulation and study design do not isolate the contribution of PQQ [52].

### 6.2. Photoaging and Pollution-Associated Skin Stress

Because PQQ can influence ROS, mitochondrial function, and inflammasome signaling, it may be relevant to photoaging and urban-exposure formulations. The most defensible early clinical model would not be a broad anti-wrinkle trial. A better design would use a controlled stress challenge with short-term mechanistic endpoints: erythema index, transepidermal water loss, tape-strip cytokines, oxidative lipid products, mitochondrial DNA damage, and recovery kinetics. Positive mechanistic results could then justify longer studies of texture, elasticity, and pigmentation.

### 6.3. Adjunctive Use After Energy-Based Procedures

Energy-based and minimally invasive procedures trigger controlled inflammation and redox signaling needed for remodeling. A direct antioxidant effect refers to concentration- and timing-dependent chemical or enzymatic modulation of reactive species, whereas a mitochondrial effect refers to preservation of membrane potential and respiration or to slower signaling through CREB–PGC-1α, SIRT1–PGC-1α, NRF2, and TFAM. These mechanisms overlap but are not interchangeable: ROS reduction can occur without mitochondrial biogenesis, and mitochondrial adaptation can persist after the immediate oxidative phase [44,59].

PQQ could therefore be studied as a recovery adjunct after fractional laser, radiofrequency, focused ultrasound, intense pulsed light, or microneedling, but no PQQ-specific clinical trial has evaluated these combinations. After fractional laser or microneedling, barrier disruption may increase exposure and irritation risk; after nonablative radiofrequency or focused ultrasound, the barrier remains largely intact and topical penetration remains limiting. An antioxidant applied too early or at excessive concentration could also attenuate beneficial redox signaling. Initial studies should compare pretreatment, immediate, delayed, and maintenance schedules and should measure tolerability, erythema, edema, transepidermal water loss, recovery time, and objective mitochondrial or oxidative biomarkers before evaluating wrinkles or collagen remodeling.

### 6.4. Combination with Injectable Skin Boosters

Intradermal delivery could bypass the barrier and expose fibroblasts and vascular cells directly, but this is the least mature and highest-risk application. PQQ should not be added empirically to polynucleotide, hyaluronic-acid, amino-acid, exosome, or biostimulatory formulations. Compatibility, osmolality, pH, particulate formation, redox interactions, local cytotoxicity, genotoxicity, sensitization, and degradation must be tested. Injectable development would require pharmaceutical—not cosmetic—standards. Until such data exist, discussion of intradermal PQQ should remain a research hypothesis.

### 6.5. Pigmentation and Erythema

Mitochondrial redox state influences melanocyte stress and keratinocyte-melanocyte signaling, but no convincing clinical evidence currently shows that PQQ treats melasma or post-inflammatory hyperpigmentation. A murine B16 melanoma-cell study reported reduced melanogenesis and tyrosinase expression [47], but this preclinical result cannot be extrapolated to human pigment disorders. Similarly, anti-inflammatory effects may reduce erythema in selected contexts, but vascular responses are complex. Early trials should include objective colorimetry and standardized imaging and should avoid conflating reduced inflammation with direct inhibition of melanogenesis or angiogenesis.

### 6.6. Hair and Scalp Applications

Hair follicles have high energy requirements, and mitochondrial dysfunction contributes to follicular aging. PQQ could be explored in dermal papilla cells or follicular organ culture, particularly under oxidative stress. Nonetheless, evidence for hair growth is currently insufficient. Claims regarding alopecia, follicular stem cells, or increased anagen duration would be premature.

### 6.7. Oral Nutricosmetic Use

Oral PQQ is commercially available in some markets, but nutricosmetic positioning requires evidence that systemic dosing reaches skin at biologically meaningful concentrations and changes validated skin outcomes. One small study reported improvement in selected dry-skin measures after oral PQQ, but the sample was small and replication and PQQ-specific skin pharmacokinetics are lacking [49]. A randomized trial could assess 10–20 mg/day versus placebo for 12–24 weeks with safety monitoring, plasma PQQ, oxidative biomarkers, skin autofluorescence, elasticity, hydration, wrinkle imaging, and optional biopsy. Such a study should control diet, supplements, sun exposure, smoking, and concurrent procedures.

## 7. Pharmacokinetics, Bioavailability, Formulation, and Delivery Considerations

Human pharmacokinetic data are limited and relate to oral PQQ rather than skin delivery. In studies summarized by the European Food Safety Authority, a single oral dose of approximately 0.2 mg/kg produced a peak free-serum concentration near 9 nM at 2 h. During sequential 7-day dosing at 0.075, 0.15, and 0.3 mg/kg/day, free-serum concentrations reached approximately 14 nM, while only a small fraction was recovered as unchanged urinary PQQ [23,53]. Radiolabeled-mouse data suggest gastrointestinal absorption and distribution to several tissues, including a small fraction in skin, but the relevant metabolites and human cutaneous exposure remain insufficiently characterized [53,58]. These findings cannot be used to predict an effective topical or intradermal concentration.

A successful PQQ formulation must reconcile chemical stability with biological availability. The oxidized and reduced forms may interconvert, and excipients can change redox behavior. Developers should characterize identity, purity, water content, counter-ion composition, residual solvents, heavy metals, endotoxin where relevant, and degradation products. Stability testing should include light, oxygen, heat, freeze-thaw cycles, and contact with packaging. Because PQQ can chelate metals, compatibility with mineral pigments, trace elements, and metallic device components deserves attention.

### Comparison with Other Mitochondrial-Support Compounds

PQQ differs from NAD+-related strategies and coenzyme Q10 (CoQ10) in both chemical role and evidence base. PQQ is a water-soluble redox-active quinone with proposed signaling effects on CREB–PGC-1α and SIRT1–PGC-1α, whereas nicotinamide, nicotinamide riboside, and nicotinamide mononucleotide primarily support NAD+ pools and thereby influence sirtuins, poly(ADP-ribose) polymerases, and cellular energy metabolism. Topical nicotinamide has substantially more direct clinical evidence for barrier function, pigmentation, and visible aging than PQQ, while clinical skin evidence for oral NAD+ precursors remains limited [60].

CoQ10 is a lipid-soluble endogenous electron carrier in the respiratory chain and also functions as an antioxidant; topical penetration and skin-aging data are more developed than for PQQ, although formulation stability and solubility remain challenges [61,62]. Idebenone, vitamin C, vitamin E, ferulic acid, glutathione derivatives, and resveratrol act through partly overlapping redox or stress-response pathways [63,64]. PQQ should therefore be evaluated by target engagement, stability, tolerability, and controlled clinical outcomes rather than by chemical antioxidant capacity alone. Combination studies must test synergy, redundancy, and antagonism rather than assume additive benefit.

Topical bioavailability has not been established in humans. Because PQQ is highly polar, conventional creams may retain much of the dose at the surface. Franz diffusion studies using human or porcine skin should quantify intact PQQ, reduced PQQH2, and degradation products in the stratum corneum, viable epidermis, dermis, and receptor fluid [65]. Encapsulation in liposomes, polymeric nanoparticles, hydrogels, or other carriers may improve stability or localization, but each system changes release, toxicity, and regulatory requirements and none should be assumed superior without PQQ-specific comparative data.

Microneedle patches or other device-assisted systems could bypass part of the stratum corneum and deposit a predefined dose in the epidermis or superficial dermis [66]. However, no validated PQQ microneedle formulation or human cutaneous pharmacokinetic study is currently available. Sterility, mechanical reliability, dose uniformity, degradation, sensitization, and systemic exposure would need to be characterized before clinical use.

## 8. Safety, Dosing, Potential Adverse Effects, and Regulatory Issues

Available evidence supports only short-term oral use under studied conditions. The European Food Safety Authority evaluated PQQ disodium for healthy adults at a proposed maximum intake of 20 mg/day and excluded children and pregnant or lactating women from the target population [53]. Human studies have generally used 10–20 mg/day for several weeks, with some exposures up to 100 mg/day or longer durations, but sample sizes were too small and monitoring too limited to exclude rare, renal, or long-term adverse effects [53,54,55,56,57,67]. No oral dose has been validated for wrinkle reduction, pigmentation, elasticity, or procedural recovery.

Animal toxicology indicates that kidney and urinary findings are important dose-limiting considerations at high exposure. The EFSA assessment identified a 90-day no-observed-adverse-effect level of 100 mg/kg/day for the evaluated PQQ disodium product and noted renal or urinary abnormalities at higher doses in some studies [53]. A separate 2022 product-specific assessment reported no treatment-related adverse effects at up to 600 mg/kg/day in a 90-day rat study, but formulation, study design, and sponsorship differed and these findings should not be interpreted as a human aesthetic dose [67]. The available data do not define safety for chronic use, renal impairment, medication interactions, pregnancy, lactation, childhood, or combined high-antioxidant supplementation.

Topical safety cannot be inferred from oral use. Standard testing should include cytotoxicity across relevant skin cells, irritation, sensitization, phototoxicity, photoallergy, ocular irritation where appropriate, and repeated-dose exposure. Because PQQ is redox-active, both antioxidant and pro-oxidant effects should be mapped across concentrations. Mitochondrial assays should include membrane potential, oxygen-consumption rate, extracellular acidification, ATP, mitochondrial ROS, mtDNA copy number, and cell survival rather than relying on a single fluorescent marker.

Injectable use requires a substantially higher evidentiary threshold. Sterility alone is insufficient. Developers must establish local tolerance, systemic exposure, hemocompatibility, genotoxicity, reproductive toxicology where indicated, and the absence of harmful degradation products. Unapproved compounding or mixing with existing injectables would create unpredictable risk and regulatory liability.

Regulatory classification will depend on jurisdiction, route, claims, and formulation. A cosmetic claim such as helping protect skin from oxidative stress differs from a drug claim of treating photoaging or accelerating wound healing. Device-assisted delivery and microneedles can create combination-product questions. Scientific publications should disclose product source, salt form, purity, funding, and conflicts of interest.

## 9. Proposed Translational Research Program

A staged program is preferable to immediate cosmetic efficacy trials. Stage 1 should define PQQ chemistry in candidate vehicles and establish concentration-response relationships in primary human keratinocytes, fibroblasts, melanocytes, endothelial cells, and adipose-derived stromal cells. Experiments should compare basal conditions with UV, blue light, particulate matter, heat, hydrogen peroxide, and procedure-relevant thermal or mechanical stress. Outcomes should include viability, ATP, respiration, mitochondrial membrane potential, ROS, mtDNA damage, PGC-1α, TFAM, NRF1/2 and SIRT1 activity, inflammasome markers, SASP factors, procollagen, MMPs, and barrier-related proteins.

Stage 2 should use three-dimensional skin equivalents, ex vivo human skin, and validated animal models. These systems can evaluate penetration, spatial biology, immune responses, and healing. An important design feature is to determine whether PQQ interferes with desired remodeling after fractional laser, radiofrequency, or microneedling. Histology should assess inflammation, re-epithelialization, collagen organization, elastin, vascular changes, and pigment distribution.

Stage 3 should consist of small phase-I studies. For topical products, randomized patch testing and escalating facial-area application can establish tolerability and target engagement. For oral products, existing supplement doses provide a starting point, but skin pharmacodynamic markers should be included. Injectable studies should not proceed until extensive preclinical safety data are available.

Stage 4 proof-of-concept trials should select a narrow indication. A split-face, vehicle-controlled study after fractional laser or microneedling could examine recovery time, erythema, edema, discomfort, transepidermal water loss, and standardized imaging. A separate chronic-use trial could evaluate fine wrinkles and texture over 16–24 weeks. Biopsy substudies could assess mitochondrial and matrix markers, but claims should be anchored to clinical outcomes.

Randomization, allocation concealment, blinded assessment, preregistration, prespecified primary endpoints, and correction for multiple comparisons are essential. Many aesthetic studies fail because they measure numerous exploratory outcomes in small samples and overinterpret nominal significance. PQQ research should avoid this pattern. A clear stop/go framework should require both safety and evidence of biological target engagement before larger trials.

## 10. Limitations of the Current Evidence

This review is narrative rather than systematic and did not include formal risk-of-bias scoring or meta-analysis. Although a structured search and reference screening were performed, restriction to English-language literature and heterogeneity in terminology, PQQ salt forms, doses, routes, models, and outcomes may have led to missed studies or selective emphasis. Publication bias is also possible.

More importantly, the evidence base is intrinsically limited. Most mechanistic data arise from non-cutaneous cells or organs; skin studies are few; Bmi-1 models represent accelerated genetic aging; wound-healing models are not equivalent to aesthetic procedural recovery; and the available human studies are small, short, or confounded by multi-ingredient formulations. Cutaneous pharmacokinetics, dose-response relationships, long-term safety, and direct comparisons with established antioxidants are lacking. Consequently, this review can establish biological plausibility and research priorities, but it cannot determine clinical efficacy or a recommended dermatologic dose.

## 11. Future Directions

Future work should determine whether PQQ acts primarily by generating new mitochondria, preserving existing mitochondria, improving mitophagy, or modifying inflammatory signaling in specific skin-cell populations. Single-cell transcriptomics and spatial metabolomics could identify responsive cell types. Stable-isotope tracing may reveal whether PQQ changes substrate utilization or anabolic metabolism in fibroblasts. Mitochondrial morphology and dynamics should be assessed alongside copy number because more mitochondria are not necessarily better if quality control is impaired.

Comparative studies with NAD+ precursors, coenzyme Q10, idebenone, glutathione derivatives, and exercise-mimetic pathways could clarify PQQ’s unique value. Combination therapy may be most rational when mechanisms are complementary—for example, pairing mitochondrial protection with a proven sunscreen or barrier-repair system. Conversely, combining several redox-active agents without mechanistic testing could destabilize formulations or produce antagonistic effects.

Patient stratification may also matter. Individuals with high photoexposure, smoking history, metabolic disease, or older age may have different mitochondrial deficits. Baseline mitochondrial biomarkers could identify responders, although practical noninvasive biomarkers are not yet established. Genetic variation in antioxidant enzymes, sirtuins, or mitochondrial haplotypes may further influence response.

Finally, research should address the semantic problem of “rejuvenation.” In scientific use, the term should refer to measurable restoration toward a healthier functional state, not vague marketing. Trials must specify which mitochondrial function is restored, in which cell type, for how long, and whether that change improves a patient-relevant dermatologic endpoint.

## 12. Conclusions

PQQ has a credible mechanistic profile as a redox-active modulator of mitochondrial function. Experimental evidence supports activation of CREB-PGC-1α and SIRT1-related signaling, protection of mitochondrial membrane function, reduction of oxidative injury, and modulation of inflammatory and senescence pathways. Skin-focused studies provide early support for reduced oxidative stress, DNA damage, senescence markers, MMP expression, and UVB-induced caspase-1 release. Human skin evidence is limited to small oral and multi-ingredient topical studies that do not establish PQQ-specific aesthetic efficacy. Direct, high-quality clinical evidence in aesthetic dermatology remains insufficient.

Accordingly, PQQ should currently be viewed as an investigational mitochondrial-support strategy rather than a proven skin-rejuvenation treatment. The most promising near-term applications are carefully formulated topical products and controlled peri-procedural studies with mechanistic endpoints. Existing oral skin data require independent replication and skin-specific pharmacodynamic evidence. Intradermal use is premature without pharmaceutical development and comprehensive toxicology. A disciplined translational program can determine whether PQQ offers meaningful advantages beyond existing antioxidants and mitochondrial-targeted compounds.

## Data Availability

No new data were created or analyzed in this study. Data sharing is not applicable to this article.

## References

[1] Krutmann J., Bouloc A., Sore G., Bernard B.A., Passeron T. (2017). The skin aging exposome. J. Dermatol. Sci..

[2] Fisher G.J., Wang Z.Q., Datta S.C., Varani J., Kang S., Voorhees J.J. (1997). Pathophysiology of premature skin aging induced by ultraviolet light. N. Engl. J. Med..

[3] López-Otín C., Blasco M.A., Partridge L., Serrano M., Kroemer G. (2013). The hallmarks of aging. Cell.

[4] López-Otín C., Blasco M.A., Partridge L., Serrano M., Kroemer G. (2023). Hallmarks of aging: An expanding universe. Cell.

[5] Rinnerthaler M., Bischof J., Streubel M.K., Trost A., Richter K. (2015). Oxidative stress in aging human skin. Biomolecules.

[6] Quan T., Fisher G.J. (2015). Role of age-associated alterations of the dermal extracellular matrix microenvironment in human skin aging. Gerontology.

[7] Wang A.S., Dreesen O. (2018). Biomarkers of cellular senescence and skin aging. Front. Genet..

[8] Victorelli S., Passos J.F. (2017). Telomeres and cell senescence—Size matters not. EBioMedicine.

[9] Chowanadisai W., Bauerly K.A., Tchaparian E., Wong A., Cortopassi G.A., Rucker R.B. (2010). Pyrroloquinoline quinone stimulates mitochondrial biogenesis through cAMP response element-binding protein phosphorylation and increased PGC-1alpha expression. J. Biol. Chem..

[10] Rucker R., Chowanadisai W., Nakano M. (2009). Potential physiological importance of pyrroloquinoline quinone. Altern. Med. Rev..

[11] Stites T., Storms D., Bauerly K., Mah J., Harris C., Fascetti A., Rogers Q., Tchaparian E., Satre M., Rucker R.B. (2006). Pyrroloquinoline quinone modulates mitochondrial quantity and function in mice. J. Nutr..

[12] Misra H.S., Rajpurohit Y.S., Khairnar N.P. (2012). Pyrroloquinoline-quinone and its versatile roles in biological processes. J. Biosci..

[13] Saihara K., Kamikubo R., Ikemoto K., Uchida K., Akagawa M. (2017). Pyrroloquinoline quinone, a redox-active o-quinone, stimulates mitochondrial biogenesis by activating the SIRT1/PGC-1alpha signaling pathway. Biochemistry.

[14] Tao R., Karliner J.S., Simonis U., Zheng J., Zhang J., Honbo N., Alano C.C. (2007). Pyrroloquinoline quinone preserves mitochondrial function and prevents oxidative injury in adult rat cardiac myocytes. Biochem. Biophys. Res. Commun..

[15] Jonscher K.R., Chowanadisai W., Rucker R.B. (2021). Pyrroloquinoline-quinone is more than an antioxidant: A vitamin-like accessory factor important in health and disease prevention. Biomolecules.

[16] He K., Nukada H., Urakami T., Murphy M.P. (2003). Antioxidant and pro-oxidant properties of pyrroloquinoline quinone: Implications for its function in biological systems. Biochem. Pharmacol..

[17] Akagawa M., Nakano M., Ikemoto K. (2016). Recent progress in studies on the health benefits of pyrroloquinoline quinone. Biosci. Biotechnol. Biochem..

[18] Kano K., Mori K., Uno B., Kubota T., Ikeda T., Senda M. (1990). Voltammetric determination of acid dissociation constants of pyrroloquinoline quinone and its reduced form under acidic conditions. Bioelectrochem. Bioenerg..

[19] Mukai K., Ouchi A., Nagaoka S., Nakano M., Ikemoto K. (2016). Pyrroloquinoline quinone (PQQ) is reduced to pyrroloquinoline quinol (PQQH2) by vitamin C, and PQQH2 produced is recycled to PQQ by air oxidation in buffer solution at pH 7. Biosci. Biotechnol. Biochem..

[20] Killgore J., Smidt C., Duich L., Romero-Chapman N., Tinker D., Reiser K., Melko M., Hyde D., Rucker R.B. (1989). Nutritional importance of pyrroloquinoline quinone. Science.

[21] Steinberg F.M., Gershwin M.E., Rucker R.B. (1994). Dietary pyrroloquinoline quinone: Growth and immune response in BALB/c mice. J. Nutr..

[22] Bauerly K.A., Storms D.H., Harris C.B., Hajizadeh S., Sun M.Y., Cheung C.P., Satre M.A., Fascetti A.J., Tchaparian E., Rucker R.B. (2006). Pyrroloquinoline quinone nutritional status alters lysine metabolism and modulates mitochondrial DNA content in the mouse and rat. Biochim. Biophys. Acta.

[23] Harris C.B., Chowanadisai W., Mishchuk D.O., Satre M.A., Slupsky C.M., Rucker R.B. (2013). Dietary pyrroloquinoline quinone (PQQ) alters indicators of inflammation and mitochondrial-related metabolism in human subjects. J. Nutr. Biochem..

[24] Sreedhar A., Aguilera-Aguirre L., Singh K.K. (2020). Mitochondria in skin health, aging, and disease. Cell Death Dis..

[25] Wiley C.D., Velarde M.C., Lecot P., Liu S., Sarnoski E.A., Freund A., Shirakawa K., Lim H.W., Davis S.S., Ramanathan A. (2016). Mitochondrial dysfunction induces senescence with a distinct secretory phenotype. Cell Metab..

[26] Passos J.F., Nelson G., Wang C., Richter T., Simillion C., Proctor C.J., Miwa S., Olijslagers S., Hallinan J., Wipat A. (2010). Feedback between p21 and reactive oxygen production is necessary for cell senescence. Mol. Syst. Biol..

[27] Miwa S., Kashyap S., Chini E., von Zglinicki T. (2022). Mitochondrial dysfunction in cell senescence and aging. J. Clin. Investig..

[28] Korolchuk V.I., Miwa S., Carroll B., von Zglinicki T. (2017). Mitochondria in cell senescence: Is. mitophagy the weakest link?. EBioMedicine.

[29] Berneburg M., Plettenberg H., Medve-König K., Pfahlberg A., Gers-Barlag H., Gefeller O., Krutmann J. (2004). Induction of the photoaging-associated mitochondrial common deletion in vivo in normal human skin. J. Investig. Dermatol..

[30] Schroeder P., Gremmel T., Berneburg M., Krutmann J. (2008). Partial depletion of mitochondrial DNA from human skin fibroblasts induces a gene expression profile reminiscent of photoaged skin. J. Investig. Dermatol..

[31] Fisher G.J., Datta S.C., Talwar H.S., Wang Z.Q., Varani J., Kang S., Voorhees J.J. (1996). Molecular basis of sun-induced premature skin ageing and retinoid antagonism. Nature.

[32] Quan T., Qin Z., Xia W., Shao Y., Voorhees J.J., Fisher G.J. (2009). Matrix-degrading metalloproteinases in photoaging. J. Investig. Dermatol. Symp. Proc..

[33] Yaar M., Gilchrest B.A. (2007). Photoageing: Mechanism, prevention and therapy. Br. J. Dermatol..

[34] Krutmann J., Schikowski T., Morita A., Berneburg M. (2021). Environmentally-induced (extrinsic) skin aging: Exposomal factors and underlying mechanisms. J. Investig. Dermatol..

[35] Schikowski T., Hüls A. (2020). Air pollution and skin aging. Curr. Environ. Health Rep..

[36] Burke K.E. (2018). Mechanisms of aging and development—A new understanding of environmental damage to the skin and prevention with topical antioxidants. Mech. Ageing Dev..

[37] Cho S., Shin M.H., Kim Y.K., Seo J.E., Lee Y.M., Park C.H., Chung J.H. (2009). Effects of infrared radiation and heat on human skin aging in vivo. J. Investig. Dermatol. Symp. Proc..

[38] Liebel F., Kaur S., Ruvolo E., Kollias N., Southall M.D. (2012). Irradiation of skin with visible light induces reactive oxygen species and matrix-degrading enzymes. J. Investig. Dermatol..

[39] Coppé J.P., Desprez P.Y., Krtolica A., Campisi J. (2010). The senescence-associated secretory phenotype: The dark side of tumor suppression. Annu. Rev. Pathol..

[40] Gorgoulis V., Adams P.D., Alimonti A., Bennett D.C., Bischof O., Bishop C., Campisi J., Collado M., Evangelou K., Ferbeyre G. (2019). Cellular senescence: Defining a path forward. Cell.

[41] Victorelli S., Lagnado A., Halim J., Moore W., Talbot D., Barrett K., Chapman J., Birch J., Ogrodnik M., Meves A. (2019). Senescent human melanocytes drive skin ageing via paracrine telomere dysfunction. EMBO J..

[42] Ressler S., Bartkova J., Niederegger H., Bartek J., Scharffetter-Kochanek K., Jansen-Dürr P., Wlaschek M. (2006). p16INK4A is a robust in vivo biomarker of cellular aging in human skin. Aging Cell.

[43] Zhang C., Wen C., Lin J., Shen G. (2015). Protective effect of pyrroloquinoline quinine on ultraviolet A irradiation-induced human dermal fibroblast senescence in vitro proceeds via the anti-apoptotic sirtuin 1/nuclear factor-derived erythroid 2-related factor 2/heme oxygenase 1 pathway. Mol. Med. Rep..

[44] Gruber J.V., Holtz R. (2022). Pyrroloquinoline quinone disodium (PQQ2Na) has an NLRP inflammasome-induced caspase-1 release influence in UVB-irradiated but not ATP-treated human keratinocytes but has no influence in increasing skin cell mitochondrial biogenesis in either human keratinocytes or fibroblasts. Clin. Cosmet. Investig. Dermatol..

[45] Li J., Liu M., Liang S., Yu Y., Gu M. (2022). Repression of the antioxidant pyrroloquinoline quinone in skin aging induced by Bmi-1 deficiency. Biomed. Res. Int..

[46] Gao Y., Kamogashira T., Fujimoto C., Iwasaki S., Yamasoba T. (2022). Pyrroloquinoline quinone (PQQ) protects mitochondrial function of HEI-OC1 cells under premature senescence. npj Aging.

[47] Sato K., Toriyama M. (2009). Effect of pyrroloquinoline quinone (PQQ) on melanogenic protein expression in murine B16 melanoma. J. Dermatol. Sci..

[48] Li B., Yang X.M., Zhou X.M., Huang Y.Q. (2025). Effect of pyrroloquinoline quinone on skin aging in Bmi-1 KO mice and underlying mechanisms. PLoS ONE.

[49] Nakano M., Kamimura A., Watanabe F., Kamiya T., Watanabe D., Yamamoto E., Fukagawa M., Hasumi K., Suzuki E. (2015). Effects of orally administered pyrroloquinoline quinone disodium salt on dry skin conditions in mice and healthy female subjects. J. Nutr. Sci. Vitaminol..

[50] Mohamad Ishak N.S., Kikuchi M., Ikemoto K. (2024). Dietary pyrroloquinoline quinone hinders aging progression in male mice and D-galactose-induced cells. Front. Aging.

[51] Davoodi F., Mohammadi R., Asri-Rezaei S., Behfar M., Dezfoulian O., Raisi A. (2025). Effects of pyrroloquinoline quinone (PQQ) on skin wound healing in mice. Surgery.

[52] Draelos Z.D., McDaniel D.H., Yoelin S., Pot S., Sotir O., Nelson D.B. (2023). Evaluation of a new, advanced antioxidant containing topical allyl pyrroloquinoline quinone: Analysis of antioxidant properties and visible effects in subjects with facial photodamage. J. Clin. Aesthet. Dermatol..

[53] EFSA Panel on Dietetic Products, Nutrition and Allergies (NDA) (2017). Safety of pyrroloquinoline quinone disodium salt as a novel food pursuant to Regulation (EC) No 258/97. EFSA J..

[54] Hwang P.S., Machek S.B., Cardaci T.D., Wilburn D.T., Kim C.S., Suezaki E.S., Willoughby D.S. (2020). Effects of pyrroloquinoline quinone (PQQ) supplementation on aerobic exercise performance and indices of mitochondrial biogenesis in untrained men. J. Am. Coll. Nutr..

[55] Tamakoshi M., Suzuki T., Nishihara E., Nakamura S., Ikemoto K. (2023). Pyrroloquinoline quinone disodium salt improves brain function in both younger and older adults. Food Funct..

[56] Nakano M., Kawasaki Y., Suzuki N., Takara T. (2015). Effects of pyrroloquinoline quinone disodium salt intake on the serum cholesterol levels of healthy Japanese adults. J. Nutr. Sci. Vitaminol..

[57] Ikemoto K., Mohamad Ishak N.S., Akagawa M. (2024). The effects of pyrroloquinoline quinone disodium salt on brain function and physiological processes. J. Med. Investig..

[58] Yan T., Nisar M.F., Hu X., Chang J., Wang Y., Wu Y., Liu Z., Cai Y., Jia J., Xiao Y. (2024). Pyrroloquinoline quinone (PQQ): Its impact on human health and potential benefits. Curr. Res. Food Sci..

[59] Charrier D., Cerullo G., Carpenito R., Vindigni V., Bassetto F., Simoni L., Moro T., Paoli A. (2024). Metabolic and biochemical effects of pyrroloquinoline quinone (PQQ) on inflammation and mitochondrial dysfunction: Potential health benefits in obesity and future perspectives. Antioxidants.

[60] Boo Y.C. (2021). Mechanistic basis and clinical evidence for the applications of nicotinamide (niacinamide) to control skin aging and pigmentation. Antioxidants.

[61] Zmitek K., Pogacnik T., Mervic L., Zmitek J., Pravst I. (2017). The effect of dietary intake of coenzyme Q10 on skin parameters and condition. Biofactors.

[62] Lain E.T., Agrawal N., Ruvolo E., Weise J.M., Callender V.D. (2024). The role of coenzyme Q10 in skin aging and opportunities for topical intervention: A review. J. Clin. Aesthet. Dermatol..

[63] Papaccio F., D’Arino A., Caputo S., Bellei B. (2022). Focus on the contribution of oxidative stress in skin aging. Antioxidants.

[64] Pullar J.M., Carr A.C., Vissers M.C.M. (2017). The roles of vitamin C in skin health. Nutrients.

[65] Prausnitz M.R., Langer R. (2008). Transdermal drug delivery. Nat. Biotechnol..

[66] Ita K. (2015). Transdermal delivery of drugs with microneedles—Potential and challenges. Pharmaceutics.

[67] Shiojima Y., Deshmukh N., Moriyama H., Soman Y., Nalge P., Randhe M., Kanhere J., Karmarkar A., Bagchi M., Bagchi D. (2022). Safety assessment of a novel, dietary pyrroloquinoline quinone disodium salt (mnemoPQQ^®^). Toxicol. Mech. Methods.

